# Temporal speed prevails on interval duration in the SNARC-like effect for tempo

**DOI:** 10.3758/s13414-023-02816-z

**Published:** 2023-11-20

**Authors:** Alberto Mariconda, Mauro Murgia, Matteo De Tommaso, Serena Mingolo, Tiziano Agostini, Valter Prpic

**Affiliations:** 1 Department of Life Sciences, University of Trieste, Trieste, Italy; 2 Department of Psychology, University of Milan Bicocca, Milan, Italy; 3 Department of Philosophy and Communication Studies, University of Bologna, Bologna, Italy; 4 Institute for Psychological Sciences, De Montfort University, Leicester, UK

**Keywords:** Temporal Processing, Music cognition, Sound recognition

## Abstract

The Spatial-Numerical Association of Response Codes (SNARC) effect is evidence of an association between number magnitude and response position, with faster left-key responses to small numbers and faster right-key responses to large numbers. Similarly, recent studies revealed a SNARC-like effect for tempo, defined as the speed of an auditory sequence, with faster left-key responses to slow tempo and faster right-key responses to fast tempo. In order to address some methodological issues of previous studies, in the present study we designed an experiment to investigate the occurrence of a SNARC-like effect for tempo, employing a novel procedure in which only two auditory beats in sequence with a very short interstimulus interval were used. In the “temporal speed” condition, participants were required to judge the temporal speed (slow or fast) of the sequence. In the “interval duration” condition, participants were required to judge the duration of the interval between the two beats (short or long). The results revealed a consistent SNARC-like effect in both conditions, with faster left-hand responses to slow tempo and faster right-hand responses to fast tempo. Interestingly, the consistency of the results across the two conditions indicates that the direction of the SNARC-like effect was influenced by temporal speed even when participants were explicitly required to focus on interval duration. Overall, the current study extends previous findings by employing a new paradigm that addresses potential confounding factors and strengthens evidence for the SNARC-like effect for tempo.

## Introduction

In cognitive psychology the relationship between space and numbers has been largely studied in the last 30 years. The scientific literature has documented widely that people tend to associate small numbers to the left and large numbers to the right, along a left-to-right-oriented mental number line (Restle, 1970). Dehaene et al. (1993) first demonstrated the existence of this spatial-numerical relationship with the so-called Spatial-Numerical Association of Response Codes (SNARC) effect. The SNARC-effect consists of faster left-key responses to small numbers (e.g., 1) and faster right-key responses to large numbers (e.g., 9). Since this seminal work by Dehaene et al. (1993), several studies have focused on different aspects of the SNARC effect, such as flexibility and context dependence (Bächtold et al., 1998; Cipora et al., 2018; Mingolo et al., 2021), and the role of order and magnitude information (Pitt & Casasanto, 2020; Prpic et al., 2021).

Numerous studies have documented that the SNARC effect is not limited to symbolic numerals, indeed it has also been found with non-symbolic numerosity (Cutini et al., 2019; Nuerk et al., 2005; but see also Prpic et al., 2023). Moreover, SNARC-like effects were also found with non-numerical stimuli across different modalities. In the visual modality, SNARC-like effects were found with perceptual magnitudes like the size of pictorial figures (Prpic et al., 2020; Ren et al., 2011), luminance (Fumarola et al., 2014), and angle magnitude (Fumarola et al., 2016), but also with more complex stimuli, such as different features of facial displays (Dalmaso, Schnapper et al., 2022a, Dalmaso, Vicovaro, et al., 2022b; Dalmaso & Vicovaro, 2021; Holmes et al., 2019; Holmes & Lourenco, 2011; see also Baldassi et al., 2021, and Fantoni et al., 2019, for an alternative explanation to SNARC-like effects for facial displays). Particularly important for our work are studies that investigate SNARC-like effects for musical stimuli. While only a small number of studies investigated music notation (Ariga & Saito, 2019; Fumarola et al., 2020; Prpic et al., 2016), most studies focused on the auditory modality, reporting SNARC-like effects for loudness (Bruzzi et al., 2017; Hartmann & Mast, 2017) and pitch (Lega et al., 2020; Lidji et al., 2007; Pitteri et al., 2017; Prpic & Domijan, 2018; Rusconi et al., 2006).

Like other numerical and non-numerical magnitudes, time can be spatially represented and elicit a SNARC-like effect; indeed, accumulating evidence suggest that time, similar to numbers, is cognitively represented along a mental timeline (MTL) oriented from left to right (Bonato et al., 2012). This interaction between time and space can be reflected in a left response advantage for early stimuli and a right response advantage for late stimuli (i.e., target stimuli that, following a periodic sequence of auditory stimuli, could occur either earlier or later than their expected timing; Ishihara et al., 2008; Mariconda et al., 2022). Moreover, left/right response advantages were found for short/long durations (Vallesi et al., 2008); similar spatial associations were also found for the vertical and diagonal axes (for details, see Dalmaso, Schnapper et al., 2022a, Dalmaso, Vicovaro, et al., 2022b, Topić et al., 2022).

Among the great variety of time-related aspects there is tempo, which is the speed of an auditory sequence and is commonly measured in beats per minute (bpm). De Tommaso & Prpic (2020) hypothesized that tempo can be spatially represented along the horizontal axis and, similar to other temporal information, can elicit a SNARC-like effect. In their work, the authors conducted three experiments in which they asked participants to listen to a reference sequence of beats and judge whether a subsequent target sequence was slower or faster than the reference, using lateralized response keys. In these experiments, full tempo range (40, 80, 160, 200 bpm; Experiment 1), slow tempo range (40, 56, 88, 104 bpm; Experiment 2), and fast tempo range (133, 150, 184, 201 bpm; Experiment 3) were investigated. The results revealed a SNARC-like effect only for fast tempos in Experiment 3, leading the authors to conclude that slow and fast tempos may be perceived cognitively in a different way; in particular, only fast tempos with 133 bpm or more might be associated with space.

### Methodological issues in the SNARC-like effect for tempo

Recently, Wood et al. (2021) raised methodological concerns regarding the study's design and its potential impact on the results of De Tommaso & Prpic (2020). Firstly, Wood et al. noted that participants apparently violated the Weber-Fechner law, since they were faster when discriminating fast tempos (the mean of absolute response times (RTs) was approximately 900 ms) than when discriminating slow tempos (the mean of absolute RTs was approximately 1,400 ms). Indeed, according to the Weber-Fechner law, it should be easier to discriminate the same delta of bpm with slow tempos (low number of bpm) than with fast tempos (high number of bpm). Consequentially, a pattern opposite to the Weber-Fechner law (i.e., those shown in the results of the study by De Tommaso and Prpic) could be explained if participants focused on the duration of the interval rather than on tempos. Namely, it should be easier to discriminate the same delta of bpm with a short duration of the intervals (fast tempos) than with a long duration of the intervals (slow tempos). Moreover, participants could complete the task even before listening to the second or third beat of the sequence (anticipatory responding), especially when judging the slowest tempos (e.g., for 40 and 56 bpm); this reinforced the idea that the judgment was based on interval duration rather than on tempos. An alternative explanation for the different patterns of RTs between slow and fast tempos is that participants had to spend more time in the slow condition to gain enough information to complete the task. Probably both factors (i.e., the focus on interval duration and the need for more time for gaining information) caused slower RTs for the slow tempo stimuli.

Another point highlighted by Wood et al. (2021) regards the lack of spatial associations for slow tempos, which might be due to two possible conflicting effects. Indeed, with slow tempos, the interstimulus interval might assume a higher relevance and participants might have focused on interval duration rather than on temporal speed; in other words, instead of judging temporal speed (slow or fast), they might have evaluated the temporal duration of the interval between beats (short or long). In this case, short intervals should be associated to the left and long intervals to the right (Conson et al., 2008; Vallesi et al., 2008), namely the opposite expectations to the associations for temporal speed (i.e., slow-left and fast-right, as in De Tommaso & Prpic, 2020; Exp. 3). According to Wood et al. (2021), it is possible that these two opposite patterns prevented any spatial association from occurring with slow tempos in the study by De Tommaso & Prpic (2020).

Furthermore, previous literature did not discuss a possible confound between tempo and numerosity of auditory beats (for recent evidence on common mechanisms for numerosity and temporal processing, see Fortunato et al., 2023). Indeed, when participants judge fast tempo stimuli, they are exposed to a larger number of auditory beats compared to when they judge slow tempo stimuli. Therefore, it is possible that the spatial association detected in the fast tempo range (De Tommaso & Prpic, 2020; Exp. 3) was actually due to the smaller number of beats for the relatively slower tempos, and to the larger number of beats for the relatively faster tempos. Thus, it cannot be excluded that the stimuli with smaller numbers were associated with the left and those with larger numbers were associated with the right, according to the SNARC effect for non-symbolic numerals (Cutini et al., 2019; Nemeh et al., 2018; Nuerk et al., 2005).

To sum up, the SNARC-like effect for tempo is still an unclear phenomenon that needs further investigation. The main objective of the present study is to investigate this phenomenon, taking into consideration the three methodological issues described above: (1) the anticipatory responding, (2) the focus on temporal speed or on interval duration, and (3) the confound between tempo and numerosity.

### The present study

At first, to address the methodological issue of anticipatory responding, we conducted a pilot study, in which we “turned the beat around” by presenting the target tempo before the reference, as suggested in Wood et al.'s (2021) commentary. However, the results indicated that the task was too difficult and led to an excessive number of errors, as a near-to-chance level of performance was observed (see Appendix). We then used a different approach, which implies a memory-based judgment. Specifically, we used only two beats in sequence with a very short interstimulus interval (to ensure the complete encoding of the target sequence) without employing a reference. In this way, the use of a very short interstimulus interval would address the problem of anticipatory responding (issue 1) while the presence of only two beats, both for the relatively fast and slow tempos, would address the confound between tempo and numerosity (issue 3). Clearly by using this approach it is not possible to investigate stimuli in the slow temporal range; however, it will be possible to address these methodological concerns within the fast temporal range. By employing this novel paradigm, we designed two conditions (a “temporal speed” and an “interval duration” condition). In the temporal speed condition participants were asked to judge the temporal speed of the two beats in sequence (slow or fast). In the interval duration condition participants were required to judge the duration of the interval between the two beats (short or long) of the same stimuli. This allowed us to disambiguate the effect of the focus on temporal speed or on interval duration (issue 2) in spatial associations for tempo.

In the temporal speed condition we expected faster left-hand responses for slow tempo and faster right-hand responses for fast tempo, similar to the study by De Tommaso & Prpic (2020). In the interval duration condition, one of three possible outcomes can emerge: (1) the effect is driven by interval duration, determining a spatial association with a direction opposite to the previous study (De Tommaso & Prpic, 2020); (2) spatial associations are driven by temporal speed, determining a spatial association in line with the previous study (De Tommaso & Prpic, 2020); (3) interval duration conflicts with the processing of temporal speed, thus determining a null effect.

## Method

### Participants

The sample size was calculated using the software G*Power, setting power = .80 and α = .05 for a repeated-measures ANOVAs main effect, and a small/medium effect size (f = .20), based on previous research (De Tommaso & Prpic, 2020; Mariconda et al., 2022). The result was a required sample size of 36 participants. To ensure that a sufficient number of participants could be considered for the analyses, we tested 45 participants (M = 9, F = 36; M_age_= 22.13 years, SD = 2.84 years).

All participants were Italian university students (three of them were left-handed and two were ambidextrous), and their reading/writing direction was left-to-right. All participants reported having normal hearing and normal or corrected-to-normal vision, and that their psychophysiological state was not affected by alcohol consumption or insufficient sleep in the previous 24 h (Murgia et al., 2020). Participants were students from the University of Trieste and received credits for their participation in the study. The experiment was conducted in accordance with the ethical standards established by the Declaration of Helsinki and was approved by the University of Trieste Ethics Committee. Written informed consent was obtained from each participant before data collection.

### Apparatus and stimuli

The experiment was designed and controlled by the Psychopy software version 3.0 (Peirce, 2007). The experiment was run with an HP PC with Intel Core i7 11th generation (RAM: 16 Gb) on a 32-in. MSI monitor (Optix Mag 322CR, 180 hz). Participants’ responses were collected using a five-button serial response box. Sony MDR-XB950/B headphones were employed to provide participants with stimuli; the volume was constant and set at a comfortable level for participants.

Two different sequences, namely slow tempo (corresponding to long interval duration) and fast tempo (corresponding to short interval duration), each of them composed of two auditory beats, were used as stimuli; each beat was a sinusoidal waveform consisting of a 1,000-Hz tone with a duration of 120 ms. The two sequences were identical, except for the duration of the interstimulus interval: in the slow tempo it had a duration of 100 ms, while in the fast tempo it lasted for 30 ms. Moreover, a third stimulus consisting of a single beat was used as a “no-go” condition. Before each sequence, a fixation cross was presented for 1,500 ms. Stimulus loudness was set at a comfortable level and was equal for all stimuli and all participants.

### Procedure

The experiment took place in a quiet laboratory in which participants were tested individually. Participants were asked to sit comfortably in front of the response box and the screen to read the instructions; their bodies were aligned with the response box’s midpoint.

Before starting the experiment, participants were explicitly informed which sequence was considered slow (long) and which was fast (short). Accordingly, they were exposed to the sequences to familiarize themselves with them and to make sure they could classify them correctly. In the experiment, participants were required to judge whether the temporal speed of each sequence was slow or fast (temporal speed condition) or whether the duration of the interstimulus interval was short or long (interval duration condition) by pressing one of the two response buttons as accurately and quickly as possible. An additional “no-go” condition, without the second beat, was added to ensure that participants waited for the entire sequence and maintained the focus on task. It is noteworthy that as opposed to De Tommaso & Prpic (2020), no reference stimulus was employed.

The temporal speed condition consisted of two blocks where the response mapping was counterbalanced among participants (A-B or B-A). In detail, in block A (congruent condition) the left key was assigned to the slow sequence and the right key was assigned to the fast sequence; in block B (incongruent condition) the response mapping was the opposite, namely “left-fast” and “right-slow.” A practice session and an experimental session were performed in each block. In each practice session participants performed ten trials (four slow, four fast, and two no-go), and received feedback on their RTs and accuracy (“Correct!”, “Wrong!”). In each experimental session, participants performed 50 trials (20 slow, 20 fast, and ten no-go) presented in random order, and received feedback only for wrong responses. Similarly, the interval duration condition consisted of two counterbalanced blocks (C-D or D-C). In Block C (congruent condition) the left key was assigned to the short interval duration and the right key was assigned to the long interval duration; in Block D (incongruent condition), this stimulus-response mapping was reversed (“left-long” and “right-short”). As in the temporal speed condition, participants performed ten trials (four short, four long, and two no-go) in the practice session and 50 trials (20 short, 20 long, and ten no-go) in the experimental sessions, in each block.

All participants were engaged in both conditions, thus they performed four blocks in total; the order of temporal speed and interval duration conditions was counterbalanced among participants. The experiment had a total duration of about 30 min.

### Data analysis

The no-go trials were excluded from the analyses (participants provided unwanted responses only in 2.88% of no-go trials in the temporal speed condition and 4% of no-go trials in the interval duration condition; this confirmed that they performed the tasks accurately). Moreover, the incorrect responses (5.33% in the temporal speed condition and 6.33% in the interval duration condition) and outliers (0.82% in the temporal speed condition and 1% in the interval duration condition) were excluded from the analyses. A response was considered as an outlier if the RT was shorter than 120 ms or longer than the average RTs of each participant plus 3 standard deviations (same criteria as Ishihara et al., 2008). In addition, we excluded any participants with over 50% of missing values in at least one cell of the design (e.g., left responses with the slow stimulus). Overall, two participants (4.4%) were removed due to this exclusion criteria. No analysis was conducted on accuracy rates due to the low number of errors.

Preliminarily, the difference between right-hand and left-hand RTs was computed [dRT = RT (right hand) - RT (left hand)] for each stimulus in each condition. Therefore, a 2 x 2 x 2 mixed-design analysis of variance (ANOVA) was performed on dRTs, with the following factors: Condition (temporal speed vs. interval duration) x Tempo (slow vs. fast) x Order (temporal speed - interval duration vs. interval duration - temporal speed); the former two variables were within subjects, the latter between subjects. Moreover, two paired-samples *t*-test were conducted to compare the dRTs for fast and slow stimuli, both in the temporal speed condition and in the interval duration condition. Finally, a set of one-sample *t*-tests was conducted to observe whether dRTs were significantly different from zero, for each stimulus in each condition. Data were analyzed using the software SPSS.

## Results

**Fig. 1:**
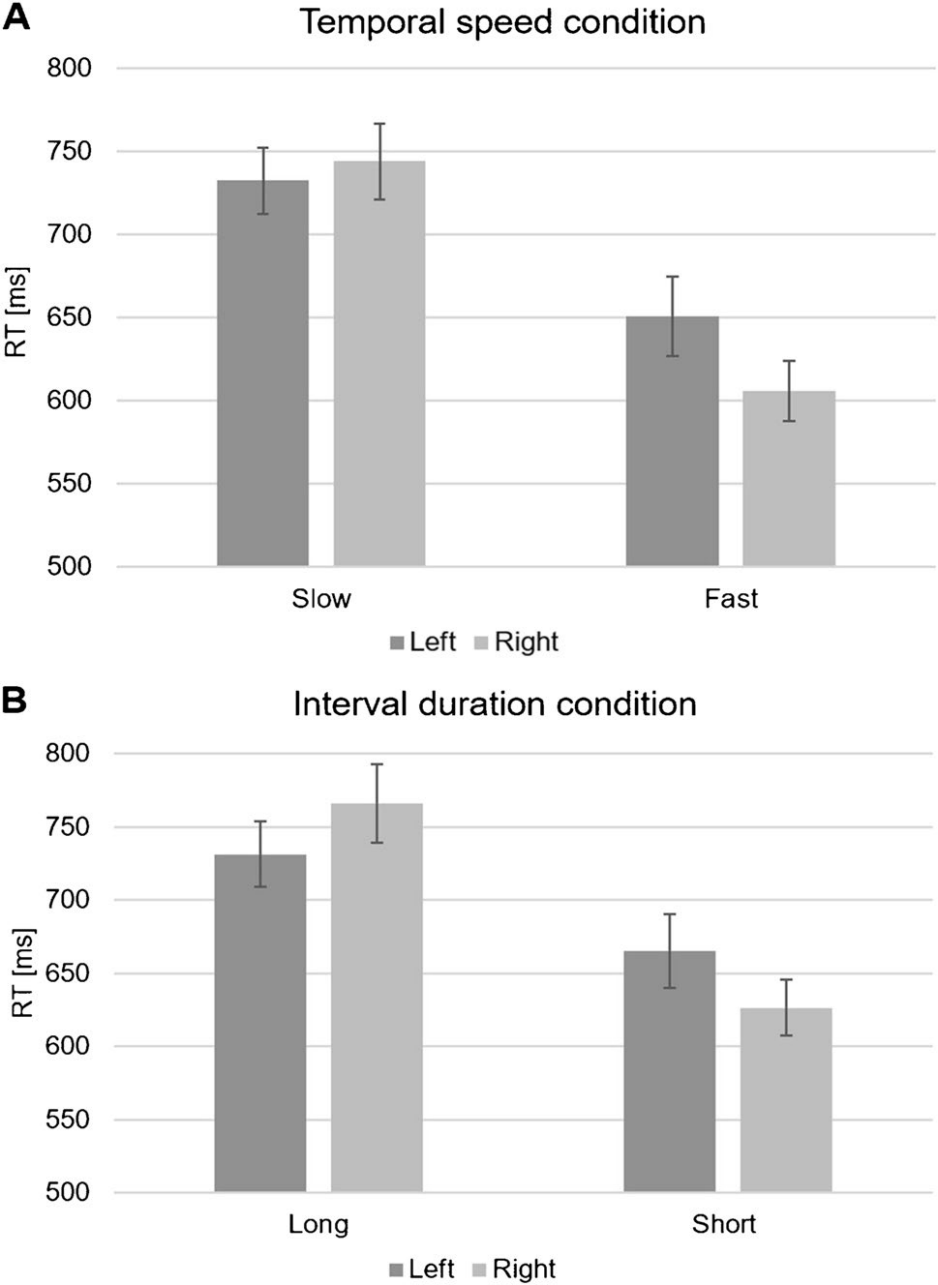
Panel **A** shows the average response times of left and right hands for slow and fast tempo stimuli in the “temporal speed” condition. Panel **B** shows the average response times of left and right hands for slow (long interval) and fast (short interval) tempo stimuli in the “interval duration” condition. Error bars indicate standard error of the mean

The analyses revealed a significant main effect for Tempo [*F*(1, 41) = 9.821; *p* < .005; *η*_*p*_^*2*^ = .193], with average higher dRTs for slow tempo stimuli and average lower dRTs for fast tempo stimuli. Figure 1 illustrates the RTs for each hand in each condition to facilitate the interpretation of this result (see also Table 1). This result indicates the presence of a SNARC-like effect for tempo, with slow tempo associated with the left and fast tempo associated with the right. Moreover, the analyses showed a significant main effect for Condition [*F*(1, 41) = 5.105; *p* < .05; *η*_*p*_^*2*^ = .111]. Conversely, no main effect (*p* = .48) or interaction with Tempo (*p* = .84), Condition (*p* = .21), or both (*p* = .94) was observed for Order; likewise, no significant Tempo x Condition interaction was found (*p* = .61), suggesting that the SNARC-like effect for tempo is similar in the two conditions.

**Table 1:** The table shows the average response times (ms) and standard deviations for slow and fast tempo stimuli with left and right hands, in both the “temporal speed” and “interval duration” conditions

Stimuli	Hand	Temporal speed condition (slow vs. fast tempo judgment)	Interval duration condition (short vs. long interval judgment)
		Mean (SD)	Mean (SD)
Slow	Left	732 (130)	731 (149)
Slow	Right	743 (148)	765 (181)
Fast	Left	650 (155)	665 (167)
Fast	Right	605 (118)	626 (127)

The consistency of the SNARC-like effect for tempo across the two conditions was further confirmed by the paired-samples *t*-tests, which showed a significant difference between dRTs for slow and for fast tempo stimuli, both in the temporal speed [*t*(42) = 2.805; *p* < .05; *d* = .313] and in the interval duration [*t*(42) = 3.209; *p* < .005; *d* = .482] conditions. Interestingly, in the temporal speed condition an asymmetric pattern was observed, with dRTs for fast tempo (M = -46 ms; SD = 95) significantly lower than zero [*t*(42) = -3.173; *p* < .005; *d* = .478], and dRTs for slow tempo not different from zero (M = 12 ms; SD = 97; *p* = .44). Conversely, a more symmetric pattern was found in the interval duration condition, with dRTs for fast tempo (M = -38 ms; SD = 98) significantly lower than zero [*t*(42) = -2.577; *p* < .05; *d* = .385], and dRTs for slow tempo (M = 35 ms; SD = 74) significantly higher [*t*(42) = 3.059; *p* < .005; *d* = .464].

## General discussion

In the present study, we investigated the occurrence of a SNARC-like effect for tempo in which we expected faster left-hand responses for slow tempos and faster right-hand responses for fast tempos, according to De Tommaso & Prpic (2020). We designed the present study considering three methodological issues that emerged from previous research: (1) the anticipatory responding, (2) the focus on temporal speed or on interval duration, and (3) the confound between tempo and numerosity. We conducted one experiment using a novel paradigm, in which only two beats in sequence with very short interstimulus interval for both fast and slow tempo were used. In the temporal speed condition participants were required to explicitly judge temporal speed, while in the interval duration condition they judged the duration of the interstimulus interval. Overall, results consistently showed faster left-hand responses to slow speed/long durations and faster right-hand responses to fast speed/short durations, suggesting that the SNARC-like effect for tempo was driven by temporal speed.

The temporal speed condition replicated findings by De Tommaso and Prpic (Exp. 3; 2020), showing a SNARC-like effect for tempo. When participants were required to judge temporal speed (slow or fast), results revealed faster left-hand responses to slow tempo and faster right-hand responses to fast tempo. The modified paradigm adopted in this experiment allowed us to address the first (anticipatory responding) and the third (confound between tempo and numerosity) methodological issues. Indeed, a paradigm with only two stimuli (fast and slow) consisting of two beats with a short interstimulus interval and no reference was used; therefore, participants completed the encoding of the target sequence before responding, thus avoiding anticipatory responding. Moreover, this paradigm with only two beats would address the confound between tempo and numerosity, as the number of beats would remain constant for both slow and fast stimuli. The results obtained with this paradigm further strengthened the observation by De Tommaso and Prpic (Exp. 3; 2020) that tempo can be spatially coded from left to right.

The interval duration condition also showed a similar SNARC-like effect when participants were required to focus on interval duration between beats (short or long). Interestingly, the spatial association pattern elicited was the same as in the temporal speed condition, suggesting that the direction of the SNARC-like effect was driven by temporal speed. Indeed, results revealed faster left-hand responses to slow tempo (long duration) and faster right-hand responses to fast tempo (short durations). This condition addresses the second methodological issue, namely the focus on interval duration (rather than on temporal speed). Indeed, even when changing the focus of the instructions, the processing of temporal speed prevails on the processing of interval duration. Therefore, it is unlikely that the processing of interval duration played a role in the lack of a spatial association for the slow tempo range in De Tommaso and Prpic (Exp. 2; 2020). Nevertheless, we should consider that this evidence was gained within a fast tempo range and thus cannot be directly extended to a slow tempo range.

Interestingly, our results differ from those of Vallesi et al. (2008) and Conson et al. (2008). Indeed, Vallesi et al. (Exp. 1; 2008) revealed that participants responded faster with their left hand when a visual stimulus lasted on the screen for 1 s (short duration), while they responded faster with their right hand when it was displayed for 3 s (long duration). Similarly, evidence of an association between left (vs. right) space and short (vs. long) durations was also found by using auditory stimuli (Conson et al., 2008). Therefore, previous works showed an opposite pattern compared to our findings in the interval duration condition (long durations/left and short durations/right). A possible explanation for these contrasting effects could be that in the works by Vallesi et al. (2008) and Conson et al. (2008) the stimuli had longer durations compared to our stimuli; in particular, Conson et al. (2008) used a longer interstimulus interval (1,000 ms) compared to ours (30 ms or 100 ms). Therefore, the stimuli used in our experiment might have made temporal speed particularly salient, thus masking the effect of interval duration.

The absence of a spatial association for interval duration (short/left, long/right, as found by Ishihara et al., 2008, and Vallesi et al., 2008) suggests the lack of automaticity of this effect. This is supported by recent evidence in the field. For example, Scozia et al. (2023a) showed that this spatial association is only found with longer time durations, suggesting that spatial interference only occurs at the later stages of processing. Moreover, another recent study by Scozia et al. (2023b) showed that spatial associations only occur when both spatial and temporal codes are jointly involved in the task. This effect gains further strength when the spatial code is response-related. Overall, our evidence supports the lack of automaticity of spatial associations for interval duration; however, it also suggests that temporal speed might be automatically associated with space since its effect emerged in the interval duration condition with very fast stimuli. Future studies should further investigate whether the spatial association for temporal speed is an automatic phenomenon.

Overall, our results seem to be in line with the ATOM model (Bueti & Walsh, 2009; Walsh, 2003), which suggests that any spatially or action-coded magnitude should be related to space. Nonetheless, our findings suggest that certain magnitudes may be preferentially associated with space compared to others, and this becomes clear only when these are put directly in contrast, such as temporal speed and interval duration in our study. Indeed, the processing of temporal speed prevails despite the focus of the instructions being changed explicitly in favor of interval duration (in the interval duration condition). Consequently, it is possible that, at least with fast stimuli (that have very a short interstimulus interval, as in our study), temporal speed becomes the most salient dimension and, consequently, it elicits a spatial association. Therefore, our study shows that explicitly stressing the instructions in favor of another dimension (i.e., interval duration) might not be sufficient to change the pattern of spatial associations.

Our evidence also seems to be in line with the polarity correspondence principles (Proctor & Cho, 2006), which posit that corresponding polarities can be conceptually associated. Since in our study we employed both dichotomous stimuli and responses, it is certainly possible that “slow” was defined as negative polarity (-) while “fast” was defined as positive (+) and, therefore, these features were associated with left (-) and right (+) responses, respectively. Interestingly, when task instructions required focusing on interval duration, temporal speed prevailed, suggesting that participants spontaneously recoded a short interval (-) as fast temporal speed (+), and a long interval (+) as slow temporal speed (-). Both magnitude-based (ATOM) and conceptual (polarity correspondence principles) accounts are possible explanations for the spatial association revealed in our study. However, our experiment was not specifically designed to differentiate between the theoretical accounts, thus there is no clear evidence for supporting one account versus the other. Future studies should investigate which theoretical approach better explains the SNARC-like effect for tempo.

Furthermore, our study revealed different absolute RTs for slow and fast tempos. This phenomenon was also observed in De Tommaso and Prpic (Exp. 2 and 3; 2020), with RTs for slow tempos being slower than those for fast tempos. Our experiment confirmed this trend, but the gap between slow and fast tempos was extensively reduced. Indeed, in De Tommaso & Prpic (2020) this gap was approximately of 500 ms (around 1,400 ms for slow temporal range vs. 900 ms for fast temporal range) compared to our experiment in which it was approximately of 100 ms (around 700 ms vs. 600 ms for slow and fast tempo stimuli, respectively). While the global reduction of RTs in our experiment (compared to the previous study) can be easily attributed to the full encoding of the stimuli before responding, it is interesting to notice that – although reduced – the gap between RTs for fast and slow stimuli still persists in our experiment. In our opinion, two explanations could clarify this gap. Since RTs were measured from the first beat, it is possible that RT gap (100 ms) was due to participants encoding the fast stimulus earlier than the slow stimulus (30 vs. 100 ms for fast/slow tempos, respectively). Alternatively, this gap could be due to the congruency between the stimuli and their semantic classification. Indeed, participants were required to classify as “fast” (or “short”) stimuli that were “extremely” fast (or short); in this case, there was a clear congruency between the stimuli and their label. Conversely, they had to classify as “slow” (or “long”) stimuli that were still “quite” fast (or short). This incongruency might have caused slower RTs for slow tempo stimuli.

A limitation of the present study is that the paradigm used in our experiment provides further evidence only regarding the processing of fast tempo, while it does not allow us to make direct inferences about slow tempos. Indeed, it is possible that the interval duration between beats would play a greater role with slow tempos because it is longer and, consequently, participants would have more time to focus on it and to process it. Therefore, interval duration might become more salient with slower tempi. Conversely, in our stimuli the interstimulus interval was particularly short, making temporal speed the relevant dimension. Unfortunately, our paradigm is not suitable to investigate this issue with slow tempos, as the gap between the relatively slow and fast stimuli should be very short to avoid anticipatory responding. Indeed, as clearly described by the Weber-Fechner law, this short gap can be easily detected within a short interval duration (fast tempo range), as in our study, but would be very hard to detect within larger interstimulus intervals (slow tempo range). Future studies should design different paradigms in order to investigate the spatial association in the slow tempo range and to assess whether this differs from the fast range, as suggested by De Tommaso & Prpic (2020).

## Conclusion

In the present study we extended previous findings by De Tommaso & Prpic (2020), taking into account three methodological issues: (1) the anticipatory responding, (2) the focus on temporal speed or on interval duration, and (3) the confound between tempo and numerosity. To do this, we developed a novel paradigm that ensured the complete encoding of the stimuli and that used a fix number of beats, thus addressing the first and third issues. Moreover, to address the second issue we asked participants to focus either on temporal speed or on interval duration. Overall, the results strengthen previous findings on the SNARC-like effect for tempo, conceptually replicating the effect by using an improved methodological paradigm. Our results suggest that the SNARC-like effect for tempo is driven by temporal speed, even when the focus of the instructions is changed explicitly in favor of interval duration.

## Appendix

This Appendix includes a brief description of the pilot study in which we “turned the beat around,” as suggested by Wood et al. (2021). The aim of this study was to address the problem of anticipatory responding highlighted in their commentary; to do so, we conducted two experiments in which we played the target tempo before the reference tempo, to ensure the complete encoding of the target sequence.

### Pilot Experiment 1

In Pilot Experiment 1 we aimed to conceptually replicate Experiment 2 of De Tommaso & Prpic (2020) by presenting the target sequence before the reference.

#### Method

##### Participants

Thirty-one participants (M = 5, F = 26; M_age_= 22.37 years, SD = 6.67 years) were recruited online for this experiment.

##### Apparatus and stimuli

The experiment was created with PsychoPy (Peirce; 2007) while Pavlovia was used to run it online. All participants used their personal computer to perform the experiment.

Five sequences of auditory beats, with five different slow tempos (40, 56, 72, 88, 104 bpm) were used as stimuli. In detail, four (40, 56, 88, 104 bpm) served as target sequence while the 72-bpm stimulus served as reference sequence.

##### Procedure

In the experiment, participants listened the target sequences played before the reference; they were required to judge whether the temporal speed of each target sequence was slower or faster than the reference sequence, by pressing a left or right response key as accurately and quickly as possible.

##### Data analysis

In this section we report only descriptive analysis because this experiment was very difficult to perform and led to an excessive number of errors. Indeed, considering that a 50% error rate means that participants responded randomly and that the mean of incorrect responses was 48.48% (SD = 30.18), we concluded that participants were not able to perform the task.

### Pilot Experiment 2

Pilot Experiment 2 was similar to Pilot Experiment 1. The only difference was that in this experiment we aimed to conceptually replicate Experiment 3 of De Tommaso & Prpic (2020), thus we only used the fast tempo range (from 133 to 201 bpm).

#### Method

##### Participants

The participants were the same as those in Pilot Experiment 1.

##### Apparatus and stimuli

The apparatus used was the same as the one used in Pilot Experiment 1. The only difference was that we used five fast tempos (133, 150, 167, 184, 201 bpm) as stimuli.

##### Procedure

The procedure was similar as the one used in the Pilot Experiment 1. The only difference was that the 133, 150, 184, 201-bpm sequences served as target stimuli while the 167-bpm sequence served as reference.

##### Data analysis

As in Pilot Experiment 1, in this section we report only descriptive analysis due to an excessive number of errors. The mean of incorrect responses was 39.31% (SD = 22.26).

##### Discussion of pilot experiments

We designed Pilot Experiments 1 and 2 following the suggestions by Wood et al. (2021). Both Pilot Experiments produced a high number of errors, which prevented to analyze RTs. This was due to the complexity of the procedure that made the task particularly difficult. Indeed, participants were required to retrieve and classify information from the target sequence, while listening to the reference. It is noteworthy that there was always a conflicting response mapping between the target sequence and the reference (they had to indicate the target sequence as “slower” while listening to a “faster” sequence, or vice versa). Considering the complexity of the task and the high number of errors we decided to try a different approach to address the issue of anticipatory responding.
